# Comment on "Risk of Sarcopenia Following Long‐Term Statin Use in Community‐Dwelling Middle‐Aged and Older Adults in Japan" by Huang et al. ‐ The Authors Reply

**DOI:** 10.1002/jcsm.70070

**Published:** 2025-09-29

**Authors:** Shih‐Tsung Huang, Fei‐Yuan Hsiao, Liang‐Kung Chen, Hidenori Arai

**Affiliations:** ^1^ Department of Pharmacy National Yang Ming Chiao Tung University Taipei Taiwan; ^2^ Center for Healthy Longevity and Aging Sciences National Yang Ming University Taipei Taiwan; ^3^ National Center for Geriatrics and Gerontology Obu Aichi Japan; ^4^ Graduate Institute of Clinical Pharmacy, College of Medicine National Taiwan University Taipei Taiwan; ^5^ School of Pharmacy, College of Medicine National Taiwan University Taipei Taiwan; ^6^ Department of Pharmacy National Taiwan University Hospital Taipei Taiwan; ^7^ Center for Geriatrics and Gerontology Taipei Veterans General Hospital Taipei Taiwan; ^8^ Taipei Municipal gan‐Dau Hospital (Managed by Taipei Veterans General Hospital) Taipei Taiwan

We appreciate Dr. Huang's interest in our study ‘Risk of Sarcopenia Following Long‐Term Statin Use in Community‐Dwelling Middle‐Aged and Older Adults in Japan’ and his thoughtful comments regarding our methodology [1]. We would like to address the concerns raised in his letter.

First, regarding the standardized mean difference (SMD) of exactly 0.00 for age after propensity score matching, we understand this observation might raise questions about potential data or calculation errors. We want to clarify that this perfect balance for age was not coincidental or erroneous, but rather the result of our methodological approach. In geriatric epidemiology research, age is a critical confounding factor that significantly influences outcomes related to muscle health and sarcopenia [2]. Therefore, in addition to our standard propensity score matching criteria, we specifically required exact age matching during the matching process. This additional constraint explains why age demonstrates a perfect SMD of 0.00, while other variables in Table 1 show varying degrees of balance after matching [3]. The absence of perfect SMDs for other variables confirms that our data processing was sound and that the matching procedure functioned as intended.

Second, regarding the exact matching procedures used when sampling with replacement in our risk set sampling methodology, we implemented a systematic approach that maintained methodological rigour while addressing the challenges inherent in longitudinal studies with time‐varying exposures. For each wave of our study, we identified all subjects who initiated statin therapy (exposed) and all concurrent nonusers (unexposed). For every exposed subject, we established a risk set comprising all unexposed individuals at that specific time point. We then calculated propensity scores using multivariable logistic regression models that incorporated all relevant baseline covariates, including demographic characteristics, health status indicators, and comorbidities.

The matching procedure specifically employed a nearest‐neighbour algorithm with a predefined calliper width (0.2 of the standard deviation of the logit of the propensity score). For each exposed subject, we selected the four unexposed subjects with the closest propensity scores within this calliper. The critical ‘with replacement’ aspect meant that after an unexposed subject was selected as a control for a particular exposed subject, that individual remained eligible for selection as a control for other exposed subjects, either within the same wave or in subsequent waves.

From an epidemiological perspective, sampling with replacement in risk set sampling offers several important advantages that enhance study validity [4]. First, it preserves the representativeness of the control population across all time points in the study. In longitudinal studies where the eligible control pool may fluctuate over time, sampling without replacement could deplete early risk sets, leading to less representative matches in later periods. Sampling with replacement ensures consistent quality of matching throughout the study timeline. Second, this approach substantially increases statistical efficiency by optimizing the use of available data, particularly in studies with limited control pools relative to exposed subjects. This efficiency is especially valuable in studies of older populations where sample sizes may be constrained by eligibility criteria, loss to follow‐up or competing risks such as mortality. Third, in the context of medication effect studies, sampling with replacement better accommodates the real‐world clinical pattern where treatment decisions are made sequentially without knowledge of future exposures. By allowing individuals to serve as controls and subsequently transition to exposed status in later waves (as illustrated in Figure S1 of our Supporting Information), this method captures the dynamic nature of treatment patterns while maintaining analytical rigour.

It is important to note that while risk set sampling itself addresses time‐dependent biases such as immortal time bias by ensuring comparisons between individuals who are at risk at the same time points, the ‘with replacement’ component specifically addresses practical implementation challenges while maintaining statistical validity. In our statistical analysis, we appropriately accounted for the potential correlation introduced by using controls multiple times to ensure valid inference.

We believe our methodological approach, as detailed in our manuscript and Supporting Information, represents a rigorous application of contemporary pharmacoepidemiologic methods designed to minimize bias and enhance internal validity when evaluating the effects of time‐varying exposures [3]. We appreciate Dr. Huang's comments, which have allowed us to further elaborate on these important methodological considerations.

## Conflicts of Interest

The authors declare no conflicts of interest.

## References

[1] J. Huang , “Comment on 'Risk of Sarcopenia Following Long‐Term Statin Use in Community‐Dwelling Middle‐Aged and Older Adults in Japan' by Huang et al.,” Journal of Cachexia, Sarcopenia and Muscle 16 (2025): e13801. 40243113 10.1002/jcsm.13801 PMC12004266

[2] L. K. Chen , L. C. Meng , L. N. Peng , et al., “Mapping Normative Muscle Health Metrics Across the Aging Continuum: A Multinational Study Pooling Data From Eight Cohorts in Japan, Malaysia and Taiwan,” Journal of Cachexia, Sarcopenia and Muscle 16, no. 1 (2025 Feb): e13731. 39971708 10.1002/jcsm.13731 PMC11839280

[3] S. T. Huang , R. Otsuka , Y. Nishita , et al., “Risk of Sarcopenia Following Long‐Term Statin Use in Community‐Dwelling Middle‐Aged and Older Adults in Japan,” Journal of Cachexia, Sarcopenia and Muscle 16 (2025): e13660. 39676595 10.1002/jcsm.13660 PMC11670166

[4] K. Ohneberg , J. Beyersmann , and M. Schumacher , “Exposure Density Sampling: Dynamic Matching With Respect to a Time‐Dependent Exposure,” Statistics in Medicine 38, no. 22 (2019): 4390–4403. 31313337 10.1002/sim.8305

